# Airway Stenosis and Tracheostomy Cannula Type as Determinants of Pharyngeal Residue in Traumatic Brain Injury Patients Using Speaking Valves

**DOI:** 10.3390/jcm15134894

**Published:** 2026-06-24

**Authors:** Burak Manay, Ramazan Güven, Alperen Şentürk, Mustafa İbas, Mehmet Nuri Elgörmüş

**Affiliations:** 1 Department of Speech and Language Therapy, Faculty of Health Sciences, Istanbul Atlas University, Istanbul 34403, Turkey; alperen.senturk@atlas.edu.tr; 2 Department of Emergency Medicine, Faculty of Medicine, Istanbul Atlas University, Istanbul 34403, Turkey; ramazan.guven@atlas.edu.tr; 3 Department of Otolaryngology, Head and Neck Surgery, Istanbul Atlas University, Istanbul 34403, Turkey; mustafa.ibas@atlas.edu.tr (M.İ.); mehmetnuri.elgormus@atlas.edu.tr (M.N.E.)

**Keywords:** airway stenosis, dysphagia, FEES, pharyngeal residue, speaking valve, traumatic brain injury, tracheostomy

## Abstract

**Background/Objectives:** Dysphagia is common in tracheostomized patients with traumatic brain injury (TBI) and may be influenced by airway pathology and tracheostomy-related factors. This study investigated whether tracheostomy cannula type is independently associated with swallowing function and pharyngeal residue after accounting for airway stenosis and clinical variables. **Methods:** This retrospective observational study included 80 tracheostomized TBI patients using a speaking valve. Participants were grouped according to cannula type (non-fenestrated vs. fenestrated). Swallowing function was evaluated using Fiberoptic Endoscopic Evaluation of Swallowing (FEES), and pharyngeal residue severity was assessed using the Yale Pharyngeal Residue Severity Rating Scale (YPRSRS). Airway stenosis severity was graded using the Cotton–Meyer classification. Multivariable ordinal logistic regression analyses were performed to identify independent predictors of pharyngeal residue. **Results:** Higher pharyngeal residue scores were observed in the fenestrated cannula group under selected conditions, particularly for 5 mL liquid (*p* = 0.039) and 5 mL semi-solid boluses (*p* = 0.004) in the vallecular region, and for 5 mL semi-solid boluses in the pyriform sinuses (*p* < 0.001). Airway stenosis grade was strongly associated with increased pharyngeal residue and reduced SpO_2_ levels (*p* < 0.001). In multivariable analyses, airway stenosis emerged as the factor most consistently associated with pharyngeal residue severity (e.g., OR = 4.909, 95% CI: 1.646–14.646, *p* = 0.004), whereas cannula type was not independently associated with most outcomes. Condition-specific associations were identified between fenestrated cannula use and pharyngeal residue in two models (vallecular residue for 5 mL semi-solid: OR = 0.354, 95% CI: 0.143–0.876, *p* = 0.025; pyriform sinus residue for 10 mL liquid: OR = 0.190, 95% CI: 0.073–0.495, *p* = 0.001); however, the direction of these associations differed from unadjusted comparisons, indicating prominent confounding by stenosis severity. **Conclusions:** FEES-estimated airway stenosis appeared to be the factor most consistently associated with pharyngeal residue severity in tracheostomized TBI patients, whereas the effect of cannula type appeared to be limited. Comprehensive airway assessment may therefore be important in dysphagia management.

## 1. Introduction

Dysphagia is a common and clinically significant complication following traumatic brain injury (TBI), contributing to increased morbidity, prolonged hospitalization, and a higher risk of aspiration-related complications [1,2]. In these patients, tracheostomy is frequently performed to secure the airway and facilitate respiratory management. However, tracheostomy itself may adversely affect swallowing physiology through mechanisms such as reduced subglottic pressure, altered airflow dynamics, and impaired laryngeal elevation [3,4].

To mitigate these effects, speaking valves are widely used to redirect expiratory airflow through the upper airway, partially restoring subglottic pressure and improving swallowing coordination [5,6]. Despite these physiological benefits, swallowing outcomes in tracheostomized patients remain highly variable, suggesting that additional factors beyond airflow restoration may play a role.

One such factor is the structural design of the tracheostomy cannula. Fenestrated cannulas aim to enhance upper airway airflow and phonation. Theoretically, they may support swallowing by facilitating subglottic pressure restoration. However, the physical presence of the fenestration may induce localized mucosal trauma and granulation, potentially counteracting these benefits [7,8]. Previous studies have reported conflicting findings regarding the effects of cannula type on swallowing function, and it remains unclear whether these effects are directly attributable to the cannula itself or are influenced by underlying airway pathology.

Airway stenosis, often resulting from prolonged tracheostomy or granulation tissue formation, represents another critical determinant of respiratory and swallowing function [8,9,10,11]. Increased airway resistance and reduced oxygenation may adversely affect swallowing efficiency and pharyngeal clearance. Although airway stenosis has been described in tracheostomized patients, its independent contribution to swallowing impairment—particularly in relation to cannula type—has not been sufficiently clarified.

Importantly, most previous studies have evaluated these factors in isolation, without accounting for potential confounding between cannula characteristics and airway pathology. This limitation makes it difficult to determine whether observed differences in swallowing outcomes are truly related to cannula type or reflect underlying airway conditions.

Therefore, the aim of this study was to investigate the effects of tracheostomy cannula type on pharyngeal residue and swallowing function in patients with TBI using speaking valves, while simultaneously evaluating the role of airway stenosis and other clinical variables. Specifically, this study sought to determine whether cannula type is independently associated with swallowing outcomes after adjusting for airway stenosis, tracheostomy duration, and demographic factors.

## 2. Materials and Methods

### 2.1. Study Design and Setting

This study was designed as a retrospective, observational, and comparative study and was conducted at Istanbul Atlas University Hospital. The study data were obtained from electronic medical records and archived FEES video recordings collected as part of routine clinical practice between November 2022 and February 2026. No additional interventions were performed on the patients, and all analyses were based on existing clinical data. This study was reported in accordance with the STROBE guidelines for observational studies.

In this study, patients with two different types of tracheostomy cannulas (non-fenestrated and fenestrated) were compared in terms of swallowing function, pharyngeal residue severity, airway stenosis graded according to the Cotton–Meyer classification, and SpO_2_ values measured during speaking valve use.

The study was approved by the Atlas University Non-Interventional Ethics Committee (Decision No. 73; Meeting No. 02; 23 February 2026; Ref. No. E-22686390-050.99-93092) and was conducted in accordance with the Declaration of Helsinki.

### 2.2. Study Population

**Figure 1:**
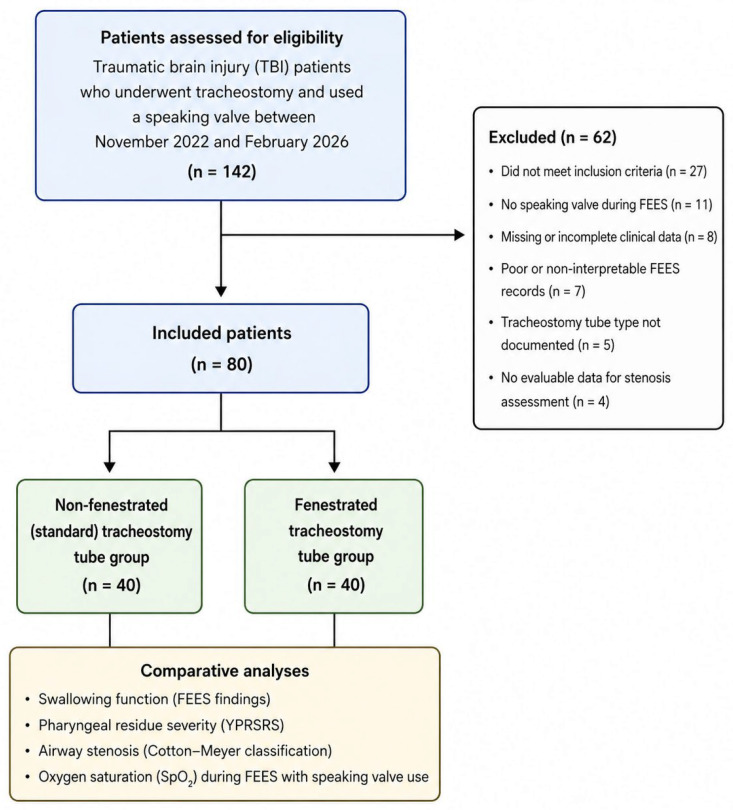
Flow diagram of patient selection and study design.

The study sample was determined through a retrospective review of the medical records of patients who met the inclusion criteria within the specified time period. A total of 80 patients diagnosed with TBI, who had undergone tracheostomy and were using a speaking valve, were included in the study. The ages of the participants ranged from 21 to 69 years. The patient selection process is illustrated in Figure 1. Patients were divided into two groups according to the type of tracheostomy cannula used: non-fenestrated and fenestrated.

The inclusion criteria were as follows: being 18 years of age or older; having a diagnosis of TBI confirmed by clinical and/or radiological findings; using a speaking valve (Passy-Muir or equivalent) at the time of assessment and during FEES evaluation; being followed at Istanbul Atlas University Hospital between November 2022 and February 2026; having available FEES recordings that included standard bolus trials (5 mL and 10 mL liquid, and 5 mL and 10 mL semi-solid [yogurt consistency]); having pharyngeal residue severity assessable using the Yale Pharyngeal Residue Severity Rating Scale (YPRSRS); having clearly documented tracheostomy cannula type in the medical records; and having SpO_2_ data recorded during FEES. In addition, patients were required to be clinically stable in the post-acute phase and suitable for swallowing assessment.

The exclusion criteria were as follows: being under 18 years of age; having a history of neurological disease other than TBI (e.g., stroke or neurodegenerative diseases); presence of head and neck tumors or structural abnormalities that could affect swallowing; FEES evaluations performed without a speaking valve; insufficient or non-interpretable FEES recordings; missing clinical or demographic data; inability to determine the tracheostomy cannula type; and records in which airway stenosis could not be evaluated.

### 2.3. Data Sources

Patients were identified through the Hospital Information Management System (HIMS) using diagnostic codes for TBI (ICD-10: S06) and dysphagia (ICD-10: R13). Demographic and clinical data of the included patients were obtained from electronic medical records and included variables such as age, gender, presence and duration of tracheostomy, type of tracheostomy cannula used (non-fenestrated/fenestrated), speaking valve use, nutritional status, and length of hospital stay.

FEES video recordings were reviewed, and parameters related to swallowing function were analyzed. Pharyngeal residue severity was assessed using the Yale Pharyngeal Residue Severity Rating Scale (YPRSRS). Airway stenosis was graded according to the Cotton–Meyer classification based on the degree of lumen narrowing observed in FEES images. All FEES recordings were independently evaluated by two experienced speech and language therapists, and inter-rater reliability was analyzed.

### 2.4. Assessment Procedures

#### 2.4.1. Fiberoptic Endoscopic Evaluation of Swallowing (FEES)

Fiberoptic Endoscopic Evaluation of Swallowing (FEES) is a reliable and bedside-applicable method used to assess swallowing function and pharyngeal clearance in tracheostomized and neurological patients. In this study, previously recorded FEES video data obtained as part of routine clinical practice were analyzed. FEES assessments were performed during the post-acute phase, defined as clinical stability permitting oral bolus trials following multidisciplinary team authorization. All FEES evaluations were conducted with the patient seated or semi-reclined at a minimum of 45 degrees. Oral intake status was not uniformly standardized across patients at the time of assessment. Some patients were receiving partial oral intake, whereas others were nil by mouth. This variability is acknowledged as a study limitation. The presence of swallowing impairment (dysphagia) was determined based on FEES findings. Pharyngeal residue amount and the efficiency of pharyngeal clearance were evaluated using the FEES recordings. Video segments corresponding to bolus trials of 5 mL and 10 mL liquid, and 5 mL and 10 mL semi-solid (yogurt consistency), were included in the analyses.

Penetration and aspiration were not evaluated within the scope of this study. In the FEES method, particularly in patients with advanced airway stenosis, limited visualization of the subglottic region may hinder the objective assessment of aspiration. Therefore, the Penetration–Aspiration Scale (PAS) was not included in the analyses.

All FEES assessments were performed while the speaking valve was in place and actively used. To minimize variability related to airflow dynamics and subglottic pressure, all patients were evaluated under comparable speaking valve conditions during the assessment. Although different speaking valve brands (e.g., Passy-Muir or equivalent) were used, their functional mechanism—allowing one-way expiratory airflow toward the upper airway—was consistent across patients. This approach allowed for a more controlled comparison of tracheostomy cannula types in terms of their potential effects on swallowing function.

#### 2.4.2. Pharyngeal Residue Assessment (YPRSRS)

YPRSRS, developed by Neubauer et al. (2015), is a five-point ordinal scale used to assess the amount of pharyngeal residue accumulated in the vallecula and pyriform sinuses during FEES [12]. The scale classifies residue severity from 1 (no residue) to 5 (severe residue) [12]. The Turkish validity and reliability of the YPRSRS were established by Atar et al. (2022), demonstrating high intra-rater and inter-rater reliability [13]. In the present study, pharyngeal residue severity was evaluated using the YPRSRS based on archived FEES video recordings.

#### 2.4.3. Airway Stenosis Assessment (Cotton–Meyer Classification)

Airway stenosis was evaluated based on the degree of lumen narrowing observed in FEES images. In our clinical setting, formal laryngotracheoscopy was not routinely performed as a standalone procedure; however, the transnasal endoscopic passage used during FEES provides direct visualization of the supraglottic and glottic airway, allowing identification of granulation tissue and luminal narrowing. Stenosis severity was classified according to the Cotton–Meyer classification based on visual estimation of percentage lumen reduction relative to the expected airway diameter [14]. According to this classification, stenosis was categorized as Grade 1 (<50% narrowing), Grade 2 (51–70% narrowing), and Grade 3 (71–99% narrowing). Cotton–Meyer grading was based on visual estimation and was used as a surrogate marker of airway narrowing rather than a definitive stenosis diagnosis. FEES video recordings were independently evaluated by two experienced speech–language therapists who were blinded to patients’ group allocation (cannula type). Inter-rater reliability was excellent across all bolus conditions (ICC 0.91–0.93). We acknowledge that visual FEES-based grading is not equivalent to bronchoscopy performed under controlled conditions, and validation against bronchoscopic or radiological imaging was not available in this dataset; this represents a limitation that should be addressed in future prospective studies.

#### 2.4.4. Oxygen Saturation (SpO_2_) Measurement

SpO_2_ values were recorded using a non-invasive pulse oximeter during FEES assessment while the speaking valve was in use. The SpO_2_ values obtained throughout the assessment period were included in the analyses.

### 2.5. Statistical Analysis

Statistical analyses were performed using IBM SPSS Statistics (Version 31.0, IBM Corp., Armonk, NY, USA). Descriptive statistics, including mean ± standard deviation (SD) for continuous variables and frequencies (%) for categorical variables, were used to summarize participant characteristics. The normality of continuous data was verified using the Shapiro–Wilk test.

Between-group comparisons (fenestrated vs. non-fenestrated cannula) were conducted using independent samples *t*-tests for normally distributed continuous variables and chi-square tests for categorical data. To evaluate the relationships between airway stenosis grade, pharyngeal residue scores (YPRSRS), and oxygen saturation (SpO_2_), Pearson correlation coefficients (r) were calculated.

To identify the independent predictors of pharyngeal residue severity, multivariable ordinal logistic regression analyses were performed for each bolus condition and anatomical region. Ordinal logistic regression was selected as the primary analytical method because YPRSRS is an ordinal outcome scale, and this approach does not require distributional assumptions regarding the dependent variable. The models included cannula type, stenosis grade (Cotton–Meyer classification), age, sex, and duration of tracheostomy as independent predictors. These variables were selected based on their clinical relevance and potential confounding effects on swallowing function. Results are reported as odds ratios (OR) with 95% confidence intervals (CI), Wald statistics, and *p*-values. Multicollinearity was assessed using tolerance and variance inflation factor (VIF); all VIF values were below 5.0, indicating acceptable collinearity. Statistical significance was set at *p* < 0.05.

The proportional odds assumption was evaluated using the Test of Parallel Lines. The assumption was satisfied only for the vallecular residue model with 10 mL semi-solid boluses (*p* = 0.234). In all other evaluable models, the test was significant (*p* < 0.05), indicating violation of the proportional odds assumption. For the pyriform sinus residue model with 10 mL semi-solid boluses, the test could not be computed because of convergence issues.

Inter-rater reliability for YPRSRS scoring was evaluated using the intraclass correlation coefficient (ICC) with a two-way random-effects model based on absolute agreement. For all analyses, a two-tailed *p*-value of <0.05 was considered statistically significant. Representative regression models were selected to illustrate the primary predictors across anatomical regions and bolus types.

## 3. Results

### 3.1. Participant Characteristics

A total of 80 patients were included, with 40 patients in each group. The mean age was higher in the non-fenestrated cannula group (50.23 ± 9.92 years) compared to the fenestrated group (44.78 ± 10.00 years). A higher proportion of advanced airway stenosis (Grade 3) was observed in the fenestrated cannula group, while the non-fenestrated group had a greater proportion of Grade 1 stenosis. Tracheostomy duration was slightly longer in the fenestrated group. Detailed characteristics are presented in Table 1.

### 3.2. Inter-Rater Reliability

Inter-rater reliability for YPRSRS scoring was excellent across all bolus conditions, with ICC values ranging from 0.91 to 0.93.

### 3.3. Comparison of Pharyngeal Residue According to Cannula Type

Group comparisons revealed higher pharyngeal residue scores in the fenestrated cannula group under selected conditions. In the vallecular region, significant differences were observed for 5 mL liquid (*p* = 0.039) and 5 mL semi-solid boluses (*p* = 0.004). In the pyriform sinus region, a significant difference was found only for 5 mL semi-solid boluses (*p* < 0.001). No significant differences were observed for other bolus conditions. Descriptive pharyngeal residue scores and SpO_2_ values according to cannula type and bolus characteristics are presented in Table 2. Statistical comparisons between groups are summarized in Table 3.

### 3.4. Correlation Analyses

Correlation analyses demonstrated strong positive associations between airway stenosis grade and pharyngeal residue scores (r = 0.756–0.921, *p* < 0.001), as well as a strong negative association between stenosis grade and SpO_2_ (r = −0.855, *p* < 0.001). Detailed correlation coefficients are presented in Table 4.

### 3.5. Effect of Airway Stenosis

Differences in pharyngeal residue and SpO_2_ across airway stenosis grades are presented in Table 5. Increasing stenosis grade was associated with progressively higher pharyngeal residue scores and lower SpO_2_ values. Statistically significant differences were observed across stenosis grades for most residue parameters, particularly in the non-fenestrated cannula group (Table 5).

### 3.6. Multivariable Regression Analyses

To evaluate the independent contributions of clinical and device-related factors to swallowing dysfunction, multiple regression models were constructed. Representative models detailing the predictors of pharyngeal residue are summarized in Table 6.

Across the majority of models, FEES-estimated airway stenosis grade emerged as the factor most consistently associated with pharyngeal residue severity (*p* ≤ 0.031 across all conditions). For instance, in the 5 mL liquid condition at the vallecula, stenosis grade was the sole significant independent predictor (OR = 4.909, 95% CI: 1.646–14.646, *p* = 0.004), whereas cannula type, age, and tracheostomy duration showed no independent association with residue levels (*p* > 0.05). This indicates that the higher residue scores initially observed in univariate comparisons were largely driven by the underlying severity of airway narrowing rather than the tracheostomy tube design itself.

However, condition-specific independent associations were also identified. For vallecular residue during 5 mL semi-solid bolus administration, fenestrated cannula use was independently significant (OR = 0.354, 95% CI: 0.143–0.876, *p* = 0.025), alongside stenosis grade (OR = 8.069, *p* < 0.001). For pyriform sinus residue during 10 mL liquid bolus administration, cannula type was also significant (OR = 0.190, 95% CI: 0.073–0.495, *p* = 0.001), with stenosis grade again being dominant (OR = 8.306, *p* < 0.001). Notably, the negative β coefficients for cannula type in both models indicate that, after adjustment for stenosis grade, fenestrated cannula use was associated with lower ordinal residue scores in these specific conditions, in contrast to the unadjusted group comparisons.

### 3.7. Effect of Bolus Characteristics

Increasing bolus consistency was associated with higher pharyngeal residue scores across both cannula groups, with higher residue levels observed in semi-solid conditions compared to liquid boluses. This pattern was consistently observed in both vallecular and pyriform sinus regions (Table 2 and Table 3). In contrast, the effect of bolus volume on pharyngeal residue did not show a consistent pattern across different conditions.

## 4. Discussion

The primary finding of this study is that FEES-estimated airway stenosis severity showed the strongest association with pharyngeal residue severity in this cohort, overshadowing the effect of cannula type in most conditions. While univariate analysis initially suggested that fenestrated cannulas were associated with increased residue, multivariable modeling revealed that this effect was largely mediated by the higher prevalence of advanced airway stenosis in that group.

Once stenosis grade was accounted for, the independent contribution of cannula type was no longer significant in most models, indicating that underlying airway pathology is the dominant driver of pharyngeal residue. This finding suggests that previously reported associations between cannula type and swallowing dysfunction may, at least in part, reflect underlying airway pathology rather than the direct effect of cannula design. The broader implication is that dysphagia in this population reflects a complex interaction between airway pathology, bolus properties, and device-related characteristics [15,16].

Although only representative models are presented, similar patterns were observed across other models, supporting the robustness of the findings.

The impact of airway stenosis on swallowing function can be explained by several physiological mechanisms. Increased airway resistance may impair respiratory–swallow coordination, reduce subglottic pressure, and negatively affect pharyngeal clearance [3,4]. In addition, the strong negative association observed between stenosis severity and SpO_2_ supports the notion that compromised respiratory function may further exacerbate swallowing inefficiency. Airway narrowing associated with granulation tissue formation is a well-recognized complication of prolonged tracheostomy and may lead to clinically significant respiratory and functional consequences [8,9,10,11,17].

The very strong correlations observed between stenosis grade and pharyngeal residue severity should be interpreted cautiously. One possible explanation is that severe airway narrowing may genuinely exert a substantial physiological influence on swallowing efficiency and pharyngeal clearance. However, because both airway stenosis grading and residue assessment were derived from the same FEES examinations, some degree of shared measurement context cannot be excluded. Although raters were blinded to cannula type and inter-rater reliability was excellent, observer-related dependencies may have contributed to the strength of the observed associations. Future studies using independent airway assessment methods such as bronchoscopy are needed to confirm these findings.

Despite the dominant role of airway stenosis, our findings also suggest that cannula type may have condition-specific associations with swallowing function. The independent association of fenestrated cannulas was identified in two models: vallecular residue for 5 mL semi-solid boluses (OR = 0.354, 95% CI: 0.143–0.876, *p* = 0.025) and pyriform sinus residue for 10 mL liquid boluses (OR = 0.190, 95% CI: 0.073–0.495, *p* = 0.001). Although these associations remained statistically significant after adjustment for airway stenosis and other covariates, their direction differed from the unadjusted group comparisons, suggesting that the apparent influence of cannula type may be closely intertwined with underlying airway characteristics. An important observation was the reversal in the direction of association for fenestrated cannulas after multivariable adjustment. In the unadjusted analyses, the fenestrated cannula group generally demonstrated higher pharyngeal residue scores. However, after adjustment for airway stenosis severity and other covariates, fenestrated cannula use was associated with lower residue severity in selected models. This pattern strongly suggests confounding and likely reflects the higher prevalence of advanced airway narrowing among patients using fenestrated cannulas. Therefore, crude comparisons between cannula groups should be interpreted cautiously and should not be viewed as evidence that fenestrated cannulas independently worsen swallowing outcomes. Taken together, these findings indicate that cannula-related factors may contribute to swallowing performance under selected bolus conditions. However, FEES-estimated airway stenosis severity remained the factor most consistently associated with pharyngeal residue severity across models. The mechanisms underlying these condition-specific associations remain unclear. Further prospective studies are needed to clarify these findings. Fenestrated cannulas are designed to facilitate airflow and phonation. Previous studies have suggested that they may also be associated with local airway changes that could indirectly influence swallowing efficiency [7,18,19]; direct assessment of such changes was not performed in the current study and this interpretation should therefore be regarded as speculative.

Previous studies have reported that tracheostomy may impair swallowing by reducing subglottic pressure and altering airflow dynamics [3,4,6]. Speaking valves have been shown to partially restore these mechanisms and improve swallowing coordination [5,6]. In the present study, all patients were evaluated under speaking valve conditions, which may have minimized variability related to airflow restoration. Therefore, the observed differences are more likely related to structural airway factors than to differences in expiratory airflow redirection.

Bolus characteristics also played an important role in swallowing outcomes. Increased bolus consistency was associated with greater pharyngeal residue. This finding is consistent with previous studies indicating that higher viscosity may reduce penetration–aspiration risk while impairing pharyngeal clearance [1,20,21,22]. These findings highlight the importance of individualized bolus modification strategies, taking into account both airway safety and swallowing efficiency.

From a clinical perspective, these findings suggest that airway evaluation—particularly the assessment of stenosis severity—should be prioritized in the management of dysphagia in tracheostomized patients. The selection of tracheostomy cannula type should not be based solely on theoretical advantages but should be considered in the context of the patient’s overall airway condition. In addition, optimizing bolus characteristics may help reduce pharyngeal residue and improve swallowing safety. Furthermore, the strong positive correlation between tracheostomy duration and stenosis grade (r = 0.826) implies that the impact of long-term cannulation on swallowing is indirect, operating primarily through the progressive development of airway narrowing over time.

An important methodological consideration is that airway stenosis severity was estimated using FEES-based visual grading rather than standard bronchoscopic or laryngotracheoscopic examination. Although inter-rater reliability was excellent and raters were blinded to group allocation, FEES-based Cotton–Meyer grading should be considered a surrogate indicator of airway narrowing rather than a validated measure of stenosis severity. Therefore, the observed associations should be interpreted as relationships between FEES-estimated airway narrowing and swallowing outcomes, rather than definitive evidence regarding anatomically confirmed stenosis severity.

This study has several limitations. The retrospective design limits causal inference and precludes the establishment of directionality between observed associations. A clinically important source of selection bias relates to cannula selection. In routine practice, cannula type is typically determined by the patient’s airway condition and phonation needs. Consequently, a systematic relationship exists between cannula selection and underlying airway severity, which multivariable adjustment can only partially address. The higher prevalence of advanced airway stenosis in the fenestrated cannula group likely reflects this clinical decision-making pattern rather than random allocation. Nevertheless, residual confounding from unmeasured between-group differences cannot be excluded. Several clinically relevant variables were unavailable in our retrospective dataset, including cuff status, ventilatory support parameters, cannula diameter, neurological severity scores at the time of assessment, sedation level, nutritional route, and rehabilitation intensity. Detailed information regarding feeding route (oral feeding, enteral feeding, or mixed feeding) was not consistently available in the retrospective dataset and therefore could not be summarized across groups. Consequently, potential differences in nutritional status and oral intake characteristics between groups could not be fully evaluated. This limitation should be considered when interpreting the findings. Future prospective studies should systematically document feeding methods to better characterize study populations and evaluate their potential influence on swallowing outcomes. These factors may independently influence swallowing physiology and represent important sources of potential residual confounding that future prospective studies should systematically document and control. Airway stenosis was graded using FEES-based visual estimation rather than formal laryngotracheoscopy or bronchoscopy, representing an important methodological limitation. Although inter-rater reliability was excellent and raters were blinded to group allocation, FEES-based grading is not directly equivalent to controlled endoscopic assessment, and validation against bronchoscopic findings was not available. The single-center design and relatively small sample size may limit generalizability. Penetration and aspiration were not evaluated due to limitations of FEES in visualizing the subglottic region, particularly in patients with advanced airway stenosis, which may limit the reliability of PAS scoring in this context [7,17]. In addition, the proportional odds assumption was not satisfied in several ordinal logistic regression models. Therefore, the regression findings should be interpreted with appropriate caution, and future studies may consider alternative modeling approaches when analyzing ordinal swallowing outcomes.

## 5. Conclusions

FEES-estimated airway stenosis appeared to be the factor most consistently associated with pharyngeal residue severity in tracheostomized patients with TBI, whereas the effect of cannula type appeared to be limited and condition-specific, primarily observed during the clearance of higher-viscosity boluses. These findings suggest that swallowing impairment in this population is largely driven by underlying airway pathology rather than device-related factors alone. From a clinical standpoint, management should prioritize comprehensive airway evaluation, particularly the assessment of stenosis severity. Cannula selection should also be individualized according to the patient’s overall airway condition. Optimization of bolus texture and consistency remains an important strategy for improving pharyngeal clearance and swallowing safety. Future prospective studies with standardized data collection and more controlled group allocation are needed to clarify causal relationships and guide clinical decision-making in this population.

## Figures and Tables

**Table 1:** Demographic and Clinical Characteristics.

Variable	Non-Fenestrated Cannula (n = 40)	Fenestrated Cannula (n = 40)
**Gender, n (%)**		
Male	21 (52.5%)	20 (50%)
Female	19 (47.5%)	20 (50%)
**Age (years), Mean ± SD**	50.23 ± 9.92	44.78 ± 10.00
**Duration of Tracheostomy (months), Mean ± SD**	5.58 ± 4.43	7.08 ± 4.76
**Grade (1-2-3), n (%)**		
Grade 1	22 (55%)	12 (30%)
Grade 2	12 (30%)	15 (37.5%)
Grade 3	6 (15%)	13 (32.5%)

Values are presented as mean ± standard deviation (SD) or n (%). Grade refers to the airway stenosis/granulation severity level.

**Table 2:** Pharyngeal Residue Severity (YPRSRS) and SpO_2_ Values According to Bolus Volume, Consistency, and Cannula Type.

Measurement		Non-Fenestrated Cannula (Mean ± SD)	Min–Max	Fenestrated Cannula (Mean ± SD)	Min–Max
**Vallecular Residue**	(5 mL Liquid)	2.70 ± 1.224	1–5	3.23 ± 1.000	2–5
	(10 mL Liquid)	2.10 ± 1.257	1–5	2.38 ± 0.774	1–4
	(5 mL Semi-solid)	2.50 ± 1.132	1–5	3.10 ± 0.632	2–4
	(10 mL Semi-solid)	1.83 ± 1.035	1–5	1.93 ± 0.572	1–3
**Pyriform Sinus Residue**	(5 mL Liquid)	2.28 ± 1.261	1–5	2.55 ± 0.552	2–4
	(10 mL Liquid)	1.70 ± 0.911	1–5	1.90 ± 0.632	1–3
	(5 mL Semi-solid)	2.05 ± 1.176	1–5	3.03 ± 0.800	2–5
	(10 mL Semi-solid)	1.73 ± 0.987	1–5	1.78 ± 0.620	1–3
**SpO_2_ (%)**		93.8 ± 4.077	83–100	93.28 ± 3.735	86–100

Values are presented as mean ± standard deviation (SD) and minimum–maximum values. YPRSRS: Yale Pharyngeal Residue Severity Rating Scale; SpO_2_: oxygen saturation. Semi-solid refers to yogurt consistency.

**Table 3:** Comparison of Pharyngeal Residue According to Bolus Volume and Consistency by Tracheostomy Cannula Type.

Variable		Cannula Type	N	Mean	SD	*p*
**Vallecular Residue**	(5 mL Liquid)	Non-fenestrated	40	2.7	1.22	0.039 *
		Fenestrated	40	3.23	1	
	(10 mL Liquid)	Non-fenestrated	40	2.1	1.25	0.242
		Fenestrated	40	2.38	0.77	
	(5 mL Semi-solid)	Non-fenestrated	40	2.5	1.13	0.004
		Fenestrated	40	3.1	0.63	
	(10 mL Semi-solid)	Non-fenestrated	40	1.83	1.03	0.594
		Fenestrated	40	1.93	0.57	
**Pyriform Sinus Residue**	(5 mL Liquid)	Non-fenestrated	40	2.28	1.26	0.210
		Fenestrated	40	2.55	0.55	
	(10 mL Liquid)	Non-fenestrated	40	1.7	0.91	0.258
		Fenestrated	40	1.9	0.63	
	(5 mL Semi-solid)	Non-fenestrated	40	2.05	1.17	<0.001 ***
		Fenestrated	40	3.03	0.8	
	(10 mL Semi-solid)	Non-fenestrated	40	1.73	0.98	0.787
		Fenestrated	40	1.78	0.62	

Values are presented as mean ± standard deviation (SD). *p* values indicate comparisons between non-fenestrated and fenestrated cannula groups. * *p* < 0.05, *** *p* < 0.001. Semi-solid refers to yogurt consistency.

**Table 4:** Correlations Between Clinical Variables, Pharyngeal Residue Severity, and SpO_2_ According to Cannula Type.

Cannula Type	Variables	Age		Grade		Duration of Tracheostomy (Months)	
		r	*p*	r	*p*	r	*p*
**Non-fenestrated**	Age	1	-	−0.637	<0.001 ***	−0.451	0.003 **
	Grade	−0.637	<0.001 ***	1	-	0.826	<0.001 ***
	Duration of Tracheostomy (months)	−0.451	0.003 **	0.826	<0.001 ***	1	-
	Vallecular residue (5 mL liquid)	−0.469	0.002 **	0.850	<0.001	0.714	<0.001 ***
	Vallecular residue (10 mL liquid)	−0.520	<0.001 ***	0.921	<0.001 ***	0.786	<0.001 ***
	Vallecular residue (5 mL semi-solid)	−0.485	0.002 **	0.822	<0.001 ***	0.657	<0.001 ***
	Vallecular residue (10 mL semi-solid)	−0.600	<0.001 ***	0.905	<0.001 ***	0.788	<0.001 ***
	Pyriform sinus residue (5 mL liquid)	−0.534	<0.001 ***	0.858	<0.001 ***	0.646	<0.001 ***
	Pyriform sinus residue (10 mL liquid)	−0.562	<0.001 ***	0.915	<0.001 ***	0.812	<0.001 ***
	Pyriform sinus residue (5 mL semi-solid)	−0.493	0.001 **	0.756	<0.001 ***	0.506	<0.001 ***
	Pyriform sinus residue (10 mL semi-solid)	−0.585	<0.001 ***	0.859	<0.001 ***	0.635	<0.001 ***
	SpO_2_ (%) during FEES (with speaking valve)	0.535	<0.001 ***	−0.855	<0.001 ***	−0.712	<0.001 ***
**Fenestrated**	Age	1	-	−0.736	<0.001 ***	−0.699	<0.001 ***
	Grade	−0.736	<0.001 ***	1	1	−0.807	<0.001 ***
	Duration of Tracheostomy (months)	−0.699	<0.001 ***	0.807	<0.001 ***	1	1
	Vallecular residue (5 mL liquid)	−0.167	0.304	0.249	0.121	0.282	0.078
	Vallecular residue (10 mL liquid)	−0.290	0.069	0.398	0.011 *	0.389	0.013 *
	Vallecular residue (5 mL semi-solid)	0.202	0.211	−0.056	0.733	−0.224	0.165
	Vallecular residue (10 mL semi-solid)	0.181	0.265	−0.388	0.013 *	−0.459	0.003 **
	Pyriform sinus residue (5 mL liquid)	−0.362	0.022 *	0.374	0.017 *	0.374	0.018 *
	Pyriform sinus residue (10 mL liquid)	−0.271	0.091	0.360	0.023 *	0.258	0.108
	Pyriform sinus residue (5 mL semi-solid)	−0.621	<0.001 ***	0.680	<0.001 ***	0.706	<0.001 ***
	Pyriform sinus residue (10 mL semi-solid)	−0.013	0.939	0.115	0.480	0.127	0.433
	SpO_2_ (%) during FEES (with speaking valve)	0.530	<0.001 ***	−0.835	<0.001 ***	−0.656	<0.001 ***

r: Pearson correlation coefficient; *p*: significance level. * *p* < 0.05, ** *p* < 0.01, *** *p* < 0.001. YPRSRS: Yale Pharyngeal Residue Severity Rating Scale. SpO_2_: oxygen saturation. Semi-solid refers to yogurt consistency.

**Table 5:** Comparison of Pharyngeal Residue Severity (YPRSRS) and SpO_2_ Across Grade Levels According to Bolus Volume, Consistency and Cannula Type.

Cannula Type	Variables	F	*p*	1–2		1–3		2–3	
				MD	*p*	MD	*p*	MD	*p*
**Non-fenestrated**	Vallecular residue (5 mL liquid)	48.30	<0.001	−1.386	<0.001 ***	−2.803	<0.001 ***	−1.417	<0.001 ***
	Vallecular residue (10 mL liquid)	104.40	<0.001	−1.485	<0.001 ***	−3.152	<0.001 ***	−1.667	<0.001 ***
	Vallecular residue (5 mL semi-solid)	38.91	<0.001	−1.356	<0.001 ***	−2.439	<0.001 ***	−1.083	0.006 **
	Vallecular residue (10 mL semi-solid)	85.92	<0.001	−1.159	<0.001 ***	−2.576	<0.001 ***	−1.417	<0.001 ***
	Pyriform sinus residue (5 mL liquid)	60.90	<0.001	−1.848	<0.001 ***	−2.682	<0.001 ***	−0.833	0.030 *
	Pyriform sinus residue (10 mL liquid)	96.67	<0.001	−1.038	<0.001 ***	−2.288	<0.001 ***	−1.250	<0.001 ***
	Pyriform sinus residue (5 mL semi-solid)	40.08	<0.001	−1.902	<0.001 ***	−1.985	<0.001 ***	−0.083	0.967
	Pyriform sinus residue (10 mL semi-solid)	55.45	<0.001	−1.333	<0.001 ***	−2.167	<0.001 ***	−0.833	0.006 **
	SpO_2_ (%) during FEES (with speaking valve)	50.86	<0.001	5.015	<0.001 ***	9.182	<0.001 ***	4.167	0.001 **
**Fenestrated**	Vallecular residue (5 mL liquid)	2.070	0.141	−0.717	0.153	−0.635	0.247	0.082	0.973
	Vallecular residue (10 mL liquid)	3.520	0.04	−0.333	0.472	−0.769	0.032 *	−0.436	0.267
	Vallecular residue (5 mL semi-solid)	4.880	0.013	−0.550	0.048 *	0.071	0.950	0.621	0.020 *
	Vallecular residue (10 mL semi-solid)	3.500	0.041	0.167	0.706	0.551	0.039 *	0.385	0.158
	Pyriform sinus residue (5 mL liquid)	3.310	0.047	−0.133	0.788	−0.513	0.049 *	−0.379	0.148
	Pyriform sinus residue (10 mL liquid)	4.860	0.013	0.083	0.927	−0.558	0.054	−0.641	0.016 *
	Pyriform sinus residue (5 mL semi-solid)	17.160	<0.001	−0.450	0.136	−1.353	<0.001 ***	−0.903	<0.001 ***
	Pyriform sinus residue (10 mL semi-solid)	1.070	0.354	−0.350	0.321	−0.186	0.735	0.164	0.765
	SpO_2_ (%) during FEES (with speaking valve)	50.710	<0.001	5.367	<0.001 ***	7.833	<0.001 ***	2.467	0.006 **

Values are presented as mean differences (MD). F: ANOVA test statistic; *p*: significance level. Pairwise comparisons (1–2. 1–3. 2–3) are reported with mean differences (MD) and *p* values. * *p* < 0.05, ** *p* < 0.01, *** *p* < 0.001. YPRSRS: Yale Pharyngeal Residue Severity Rating Scale. SpO_2_: oxygen saturation. Semi-solid refers to yogurt consistency.

**Table 6:** Multivariable Ordinal Logistic Regression Analysis for Predictors of Pharyngeal Residue Severity (YPRSRS).

Dependent Variable	Independent Variable	β	Wald	*p*	OR (95% CI)
Vallecular residue (5 mL liquid)	Age	0.019	0.425	0.514	1.019 (0.963–1.080)
	Gender	−0.060	0.019	0.891	0.942 (0.397–2.235)
	Stenosis grade	1.591	8.137	**0.004**	**4.909 (1.646–14.646)**
	Tracheostomy duration (month)	0.053	0.449	0.503	1.054 (0.903–1.232)
	Cannula type (fenestrated)	−0.455	1.077	0.299	0.634 (0.268–1.500)
Vallecular residue (10 mL liquid)	Age	0.028	0.809	0.368	1.028 (0.968–1.092)
	Gender	0.109	0.056	0.812	1.115 (0.453–2.746)
	Stenosis grade	2.368	14.544	**<0.001**	**10.677 (3.161–36.044)**
	Tracheostomy duration (month)	0.060	0.544	0.461	1.062 (0.905–1.247)
	Cannula type (fenestrated)	−0.061	0.018	0.894	0.941 (0.385–2.299)
Vallecular residue (5 mL semi-solid)	Age	0.040	1.761	0.185	1.041 (0.981–1.104)
	Gender	−0.017	0.001	0.970	0.983 (0.406–2.382)
	Stenosis grade	2.088	12.541	**<0.001**	**8.069 (2.540–25.614)**
	Tracheostomy duration (month)	−0.103	1.586	0.208	0.902 (0.769–1.059)
	Cannula type (fenestrated)	−1.039	5.041	**0.025**	**0.354 (0.143–0.876)**
Vallecular residue (10 mL semi-solid)	Age	−0.013	0.169	0.681	0.987 (0.929–1.048)
	Gender	0.002	0.000	0.997	1.002 (0.406–2.474)
	Stenosis grade	1.213	4.638	**0.031**	**3.364 (1.115–10.154)**
	Tracheostomy duration (month)	−0.064	0.618	0.432	0.938 (0.799–1.101)
	Cannula type (fenestrated)	−0.129	0.080	0.777	0.879 (0.359–2.149)
Pyriform sinus residue (5 mL liquid)	Age	−0.003	0.012	0.914	0.997 (0.939–1.058)
	Gender	0.492	1.147	0.284	1.636 (0.665–4.023)
	Stenosis grade	2.095	12.189	**<0.001**	**8.126 (2.506–26.365)**
	Tracheostomy duration (month)	−0.038	0.213	0.644	0.963 (0.820–1.131)
	Cannula type (fenestrated)	−0.073	0.026	0.872	0.930 (0.383–2.256)
Pyriform sinus residue (10 mL liquid)	Age	0.012	0.133	0.716	1.012 (0.947–1.081)
	Gender	0.351	0.478	0.489	1.420 (0.525–3.850)
	Stenosis grade	2.225	11.889	**0.001**	**9.253 (2.612–32.793)**
	Tracheostomy duration (month)	0.035	0.159	0.690	1.036 (0.874–1.226)
	Cannula type (fenestrated)	−0.083	0.027	0.870	0.920 (0.341–2.484)
Pyriform sinus residue (5 mL semi-solid)	Age	−0.029	0.866	0.352	0.971 (0.914–1.033)
	Gender	0.052	0.012	0.912	1.053 (0.421–2.633)
	Stenosis grade	2.117	12.343	**<0.001**	**8.306 (2.550–27.058)**
	Tracheostomy duration (month)	−0.005	0.004	0.953	0.995 (0.846–1.170)
	Cannula type (fenestrated)	−1.659	11.572	**0.001**	**0.190 (0.073–0.495)**
Pyriform sinus residue (10 mL semi-solid)	Age	0.001	0.001	0.974	1.001 (0.939–1.067)
	Gender	−0.729	2.106	0.147	0.482 (0.180–1.290)
	Stenosis grade	2.216	12.664	**<0.001**	**9.171 (2.705–31.067)**
	Tracheostomy duration (month)	−0.088	1.124	0.289	0.916 (0.777–1.078)
	Cannula type (fenestrated)	0.317	0.430	0.512	1.373 (0.533–3.540)

β: ordinal logistic regression coefficient; OR: odds ratio; CI: confidence interval. Bold values indicate statistical significance (*p* < 0.05). Stenosis grade was coded as Grade 1, Grade 2, and Grade 3 according to the Cotton–Meyer classification. Gender was coded as 0 = female and 1 = male. Cannula type was coded as 0 = non-fenestrated and 1 = fenestrated.

## Data Availability

The data that support the findings of this study are available from the corresponding author upon reasonable request.
